# 1-[2-(2-Oxo-1,3-oxazolidin-3-yl)eth­yl]-4-phenyl-1*H*-1,5-benzodiazepin-2(3*H*)-one

**DOI:** 10.1107/S160053681002828X

**Published:** 2010-07-21

**Authors:** Daouda Ballo, Noureddine Hamou Ahabchane, Hafid Zouihri, El Mokhtar Essassi, Seik Weng Ng

**Affiliations:** a Laboratoire de Chimie Organique Hétérocyclique, Pôle de Compétences Pharmacochimie, Université Mohammed V-Agdal, BP 1014 Avenue Ibn Batout, Rabat, Morocco; b CNRST Division UATRS, Angle Allal Fassi/FAR, BP 8027 Hay Riad, Rabat, Morocco; c Department of Chemistry, University of Malaya, 50603 Kuala Lumpur, Malaysia

## Abstract

The seven-membered ring in the title compound, C_20_H_19_N_3_O_3_, adopts a boat conformation with the two phenyl­ene C atoms representing the stern and the methyl­ene C atom the prow. The dihedral angle between the best plane through the seven-membered ring (r.m.s deviation = 0.358 Å) and the phenyl substituent is 55.8 (1)°. The two rings at either ends of the ethyl chain are staggered [N—CH_2_—CH_2_—N torsion angle = 57.5 (4)°].

## Related literature

For the background to 2,3-dihydro-1*H*-1,5-benzodiazepin-2-ones, see: Ahabchane *et al.* (1999 ▶). For a related structure, see: Ballo *et al.* (2010 ▶).
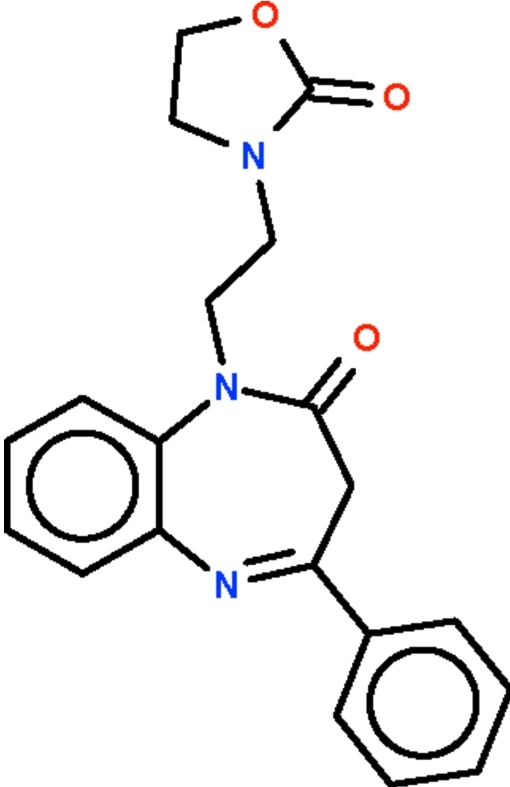


## Experimental

### 

#### Crystal data

C_20_H_19_N_3_O_3_
*M* *_r_* = 349.38
Orthorhombic, 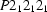

*a* = 9.0163 (5) Å
*b* = 11.6671 (6) Å
*c* = 16.2019 (8) Å
*V* = 1704.34 (15) Å^3^
*Z* = 4
Mo *K*α radiation
μ = 0.09 mm^−1^
*T* = 293 K
0.25 × 0.25 × 0.15 mm

#### Data collection

Bruker X8 APEXII diffractometer
9253 measured reflections
2053 independent reflections
1578 reflections with *I* > 2σ(*I*)
*R*_int_ = 0.039

#### Refinement

*R*[*F* ^2^ > 2σ(*F* ^2^)] = 0.036
*wR*(*F* ^2^) = 0.102
*S* = 0.90
2053 reflections
235 parameters
H-atom parameters constrained
Δρ_max_ = 0.12 e Å^−3^
Δρ_min_ = −0.16 e Å^−3^

### 

Data collection: *APEX2* (Bruker, 2008 ▶); cell refinement: *SAINT* (Bruker, 2008 ▶); data reduction: *SAINT*; program(s) used to solve structure: *SHELXS97* (Sheldrick, 2008 ▶); program(s) used to refine structure: *SHELXL97* (Sheldrick, 2008 ▶); molecular graphics: *X-SEED* (Barbour, 2001 ▶); software used to prepare material for publication: *publCIF* (Westrip, 2010 ▶).

## Supplementary Material

Crystal structure: contains datablocks global, I. DOI: 10.1107/S160053681002828X/zs2052sup1.cif

Structure factors: contains datablocks I. DOI: 10.1107/S160053681002828X/zs2052Isup2.hkl

Additional supplementary materials:  crystallographic information; 3D view; checkCIF report

## Figures and Tables

**Fig. 1.**
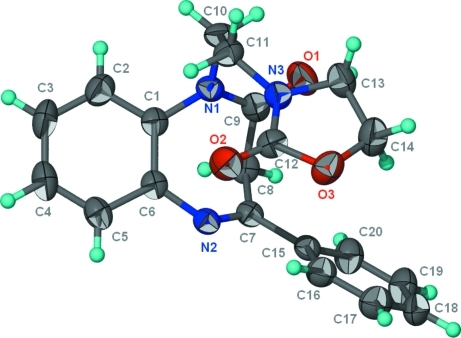
Thermal ellipsoid plot (Barbour, 2001) of the molecule of C20H19N3O3 at the 50% probability level.

## supplementary crystallographic information

### Comment

The background to the class of 2,3-dihydro-1*H*-1,5-benzodiazepin-2-ones is given in an earlier report (Ahabchane *et al.*, 1999). A recent study presented the crystal structure of 1-allyl-4-phenyl-2,3-dihydro-1*H*-1,5-benzodiazepin-2-one (Ballo *et al.*, 2010). The present study has an oxazolidin-2-onyl-ethyl group in place of the allyl group (Scheme I, Fig. 1). The principal feature is the seven-membered ring that is fused to a phenylene ring and adopts a boat-shaped conformation, two phenylene carbons representing the stern and the methylene carbon atom the prow [r.m.s deviation 0.358 Å]. The methyl carbon deviates by 0.637 Å from the best plane. The two rings at either end of the ethyl chain are staggered [N–CH_2_–CH_2_–N torsion angle, 57.5 (4)°].

### Experimental

To a solution of 4-phenyl-1*H*-1,5-benzodiazepin-2-one (2 g, 8,.4 mmol) in DMF (40 ml) was added dichloroethylamine hydrochloride (0.9 g, 8.4 mmol), potassium carbonate (3 g, 22.2 mmol) and a catalytic quantity of tetra-*n-*butylammonium bromide. The mixture was heated on a sand bath, the reaction monotired by thin layer chromatography. On completion of the reaction, the solvent was evaporated under reduced pressure. The residue was recrystallized from ethanol to afford the title compound as colorless crystals.

### Refinement

Carbon-bound H-atoms were placed in calculated positions (C–H 0.93–0.97 Å) and were included in the refinement in the riding model approximation, with *U*_iso_(H) set to 1.2–1.5*U*_eq_(C). 1486 Friedel pairs were merged in the final cycles of the refinement.

### Crystal data

**Table:**

C_20_H_19_N_3_O_3_	*F*(000) = 736
*M**_r_* = 349.38	*D*_x_ = 1.362 Mg m^−^^3^
Orthorhombic, *P*2_1_2_1_2_1_	Mo *K*α radiation, λ = 0.71073 Å
Hall symbol: P 2ac 2ab	Cell parameters from 2227 reflections
*a* = 9.0163 (5) Å	θ = 2.9–21.0°
*b* = 11.6671 (6) Å	µ = 0.09 mm^−^^1^
*c* = 16.2019 (8) Å	*T* = 293 K
*V* = 1704.34 (15) Å^3^	Prism, colorless
*Z* = 4	0.25 × 0.25 × 0.15 mm

### Data collection

**Table:**

Bruker X8 APEXII diffractometer	1578 reflections with *I* > 2σ(*I*)
Radiation source: fine-focus sealed tube	*R*_int_ = 0.039
graphite	θ_max_ = 26.7°, θ_min_ = 2.9°
φ and ω scans	*h* = −11→10
9253 measured reflections	*k* = −14→13
2053 independent reflections	*l* = −20→14

### Refinement

**Table:**

Refinement on *F*^2^	Primary atom site location: structure-invariant direct methods
Least-squares matrix: full	Secondary atom site location: difference Fourier map
*R*[*F*^2^ > 2σ(*F*^2^)] = 0.036	Hydrogen site location: inferred from neighbouring sites
*wR*(*F*^2^) = 0.102	H-atom parameters constrained
*S* = 0.90	*w* = 1/[σ^2^(*F*_o_^2^) + (0.0742*P*)^2^] where *P* = (*F*_o_^2^ + 2*F*_c_^2^)/3
2053 reflections	(Δ/σ)_max_ = 0.001
235 parameters	Δρ_max_ = 0.12 e Å^−^^3^
0 restraints	Δρ_min_ = −0.16 e Å^−^^3^

### Fractional atomic coordinates and isotropic or equivalent isotropic displacement parameters (Å^2^)

**Table:**

	*x*	*y*	*z*	*U*_iso_*/*U*_eq_	
N1	0.3905 (2)	0.62919 (16)	0.33575 (12)	0.0439 (5)	
N2	0.1611 (2)	0.45822 (16)	0.37488 (12)	0.0416 (5)	
N3	0.4287 (2)	0.61202 (17)	0.51545 (12)	0.0458 (5)	
O1	0.5951 (2)	0.51620 (17)	0.32572 (12)	0.0669 (5)	
O2	0.1947 (2)	0.60716 (17)	0.57036 (13)	0.0677 (6)	
O3	0.3700 (2)	0.48444 (16)	0.61065 (11)	0.0629 (5)	
C1	0.2372 (3)	0.6471 (2)	0.31906 (14)	0.0434 (5)	
C2	0.1935 (3)	0.7508 (2)	0.28491 (17)	0.0597 (7)	
H2	0.2650	0.8049	0.2710	0.072*	
C3	0.0461 (4)	0.7750 (2)	0.27117 (19)	0.0697 (8)	
H3	0.0181	0.8445	0.2477	0.084*	
C4	−0.0596 (3)	0.6950 (3)	0.29269 (17)	0.0646 (8)	
H4	−0.1594	0.7110	0.2841	0.078*	
C5	−0.0192 (3)	0.5927 (2)	0.32647 (15)	0.0520 (6)	
H5	−0.0921	0.5403	0.3414	0.062*	
C6	0.1301 (3)	0.5651 (2)	0.33911 (13)	0.0416 (5)	
C7	0.2709 (3)	0.39924 (19)	0.34851 (13)	0.0405 (5)	
C8	0.3658 (3)	0.4400 (2)	0.27737 (14)	0.0477 (6)	
H8A	0.4248	0.3775	0.2554	0.057*	
H8B	0.3046	0.4710	0.2335	0.057*	
C9	0.4635 (3)	0.5312 (2)	0.31346 (14)	0.0467 (6)	
C10	0.4696 (3)	0.7153 (2)	0.38443 (15)	0.0516 (6)	
H10A	0.4560	0.7899	0.3592	0.062*	
H10B	0.5748	0.6979	0.3839	0.062*	
C11	0.4161 (3)	0.7200 (2)	0.47313 (15)	0.0483 (6)	
H11A	0.4732	0.7772	0.5027	0.058*	
H11B	0.3131	0.7440	0.4737	0.058*	
C12	0.3193 (3)	0.5726 (2)	0.56469 (15)	0.0480 (6)	
C13	0.5683 (3)	0.5585 (3)	0.53446 (17)	0.0590 (7)	
H13A	0.6327	0.6096	0.5652	0.071*	
H13B	0.6190	0.5333	0.4849	0.071*	
C14	0.5192 (4)	0.4584 (3)	0.58632 (19)	0.0697 (8)	
H14A	0.5224	0.3879	0.5546	0.084*	
H14B	0.5826	0.4500	0.6343	0.084*	
C15	0.3119 (3)	0.29286 (18)	0.39276 (14)	0.0415 (5)	
C16	0.2634 (3)	0.2771 (2)	0.47373 (17)	0.0542 (6)	
H16	0.2040	0.3321	0.4989	0.065*	
C17	0.3038 (4)	0.1793 (3)	0.51664 (19)	0.0674 (8)	
H17	0.2710	0.1690	0.5706	0.081*	
C18	0.3914 (3)	0.0977 (3)	0.4807 (2)	0.0658 (8)	
H18	0.4195	0.0331	0.5104	0.079*	
C19	0.4373 (4)	0.1114 (3)	0.4010 (2)	0.0701 (8)	
H19	0.4946	0.0551	0.3759	0.084*	
C20	0.3988 (3)	0.2088 (2)	0.35785 (18)	0.0604 (7)	
H20	0.4323	0.2180	0.3040	0.072*	

### Atomic displacement parameters (Å^2^)

**Table:**

	*U*^11^	*U*^22^	*U*^33^	*U*^12^	*U*^13^	*U*^23^
N1	0.0423 (11)	0.0491 (11)	0.0402 (10)	−0.0064 (9)	−0.0003 (9)	0.0029 (9)
N2	0.0395 (11)	0.0438 (10)	0.0415 (10)	−0.0009 (9)	−0.0001 (8)	−0.0040 (9)
N3	0.0414 (11)	0.0531 (11)	0.0428 (11)	0.0005 (9)	0.0037 (9)	0.0065 (9)
O1	0.0407 (10)	0.0837 (13)	0.0761 (13)	0.0036 (10)	0.0055 (9)	0.0061 (12)
O2	0.0511 (11)	0.0706 (12)	0.0815 (13)	0.0082 (11)	0.0214 (10)	0.0049 (10)
O3	0.0706 (12)	0.0607 (11)	0.0575 (11)	0.0065 (10)	0.0181 (10)	0.0150 (9)
C1	0.0470 (13)	0.0482 (13)	0.0351 (12)	0.0017 (11)	−0.0029 (11)	−0.0027 (11)
C2	0.0711 (18)	0.0510 (14)	0.0570 (16)	0.0016 (14)	−0.0068 (15)	0.0076 (12)
C3	0.080 (2)	0.0593 (16)	0.0695 (19)	0.0250 (16)	−0.0144 (17)	0.0092 (15)
C4	0.0580 (17)	0.0736 (18)	0.0623 (17)	0.0226 (16)	−0.0112 (15)	−0.0051 (15)
C5	0.0434 (13)	0.0644 (16)	0.0483 (14)	0.0065 (12)	−0.0018 (11)	−0.0093 (13)
C6	0.0447 (12)	0.0465 (13)	0.0335 (11)	0.0032 (10)	−0.0008 (10)	−0.0061 (10)
C7	0.0412 (13)	0.0444 (11)	0.0358 (11)	−0.0024 (10)	−0.0031 (10)	−0.0058 (10)
C8	0.0558 (14)	0.0542 (13)	0.0330 (11)	0.0057 (12)	0.0038 (11)	−0.0049 (11)
C9	0.0436 (14)	0.0599 (14)	0.0367 (12)	0.0005 (12)	0.0069 (11)	0.0080 (11)
C10	0.0551 (14)	0.0547 (13)	0.0451 (13)	−0.0174 (12)	−0.0012 (12)	0.0062 (11)
C11	0.0559 (15)	0.0455 (13)	0.0435 (13)	−0.0049 (11)	0.0009 (12)	0.0003 (10)
C12	0.0544 (15)	0.0460 (13)	0.0434 (13)	0.0008 (12)	0.0077 (11)	−0.0045 (11)
C13	0.0464 (14)	0.0794 (19)	0.0513 (15)	0.0070 (13)	0.0019 (12)	0.0094 (14)
C14	0.0655 (18)	0.0788 (19)	0.0650 (17)	0.0178 (16)	0.0061 (15)	0.0164 (16)
C15	0.0388 (11)	0.0417 (11)	0.0440 (13)	−0.0032 (10)	−0.0055 (10)	−0.0049 (10)
C16	0.0557 (15)	0.0557 (14)	0.0512 (15)	−0.0024 (12)	−0.0012 (13)	0.0025 (13)
C17	0.0756 (19)	0.0734 (18)	0.0532 (16)	−0.0086 (17)	−0.0077 (15)	0.0144 (15)
C18	0.0613 (17)	0.0592 (17)	0.077 (2)	0.0008 (14)	−0.0237 (16)	0.0153 (15)
C19	0.074 (2)	0.0600 (17)	0.077 (2)	0.0218 (15)	−0.0054 (17)	−0.0025 (15)
C20	0.0643 (18)	0.0614 (16)	0.0554 (16)	0.0138 (14)	−0.0014 (14)	−0.0048 (13)

### Geometric parameters (Å, °)

**Table:**

N1—C9	1.367 (3)	C8—C9	1.501 (3)
N1—C1	1.424 (3)	C8—H8A	0.9700
N1—C10	1.463 (3)	C8—H8B	0.9700
N2—C7	1.280 (3)	C10—C11	1.517 (4)
N2—C6	1.403 (3)	C10—H10A	0.9700
N3—C12	1.350 (3)	C10—H10B	0.9700
N3—C11	1.439 (3)	C11—H11A	0.9700
N3—C13	1.439 (3)	C11—H11B	0.9700
O1—C9	1.216 (3)	C13—C14	1.505 (4)
O2—C12	1.197 (3)	C13—H13A	0.9700
O3—C12	1.350 (3)	C13—H13B	0.9700
O3—C14	1.435 (4)	C14—H14A	0.9700
C1—C2	1.387 (3)	C14—H14B	0.9700
C1—C6	1.398 (3)	C15—C20	1.377 (3)
C2—C3	1.377 (4)	C15—C16	1.395 (4)
C2—H2	0.9300	C16—C17	1.385 (4)
C3—C4	1.378 (4)	C16—H16	0.9300
C3—H3	0.9300	C17—C18	1.367 (4)
C4—C5	1.363 (4)	C17—H17	0.9300
C4—H4	0.9300	C18—C19	1.364 (5)
C5—C6	1.399 (3)	C18—H18	0.9300
C5—H5	0.9300	C19—C20	1.379 (4)
C7—C15	1.480 (3)	C19—H19	0.9300
C7—C8	1.512 (3)	C20—H20	0.9300
			
C9—N1—C1	122.7 (2)	C11—C10—H10B	109.1
C9—N1—C10	118.8 (2)	H10A—C10—H10B	107.9
C1—N1—C10	118.3 (2)	N3—C11—C10	113.2 (2)
C7—N2—C6	119.6 (2)	N3—C11—H11A	108.9
C12—N3—C11	121.5 (2)	C10—C11—H11A	108.9
C12—N3—C13	111.40 (19)	N3—C11—H11B	108.9
C11—N3—C13	123.5 (2)	C10—C11—H11B	108.9
C12—O3—C14	109.1 (2)	H11A—C11—H11B	107.7
C2—C1—C6	119.6 (2)	O2—C12—O3	122.2 (2)
C2—C1—N1	118.7 (2)	O2—C12—N3	128.1 (2)
C6—C1—N1	121.7 (2)	O3—C12—N3	109.8 (2)
C3—C2—C1	121.2 (3)	N3—C13—C14	101.5 (2)
C3—C2—H2	119.4	N3—C13—H13A	111.5
C1—C2—H2	119.4	C14—C13—H13A	111.5
C2—C3—C4	119.1 (3)	N3—C13—H13B	111.5
C2—C3—H3	120.4	C14—C13—H13B	111.5
C4—C3—H3	120.4	H13A—C13—H13B	109.3
C5—C4—C3	120.7 (3)	O3—C14—C13	105.4 (2)
C5—C4—H4	119.7	O3—C14—H14A	110.7
C3—C4—H4	119.7	C13—C14—H14A	110.7
C4—C5—C6	121.1 (3)	O3—C14—H14B	110.7
C4—C5—H5	119.4	C13—C14—H14B	110.7
C6—C5—H5	119.4	H14A—C14—H14B	108.8
C1—C6—C5	118.3 (2)	C20—C15—C16	118.1 (2)
C1—C6—N2	124.5 (2)	C20—C15—C7	122.7 (2)
C5—C6—N2	117.2 (2)	C16—C15—C7	119.2 (2)
N2—C7—C15	118.8 (2)	C17—C16—C15	119.9 (3)
N2—C7—C8	121.6 (2)	C17—C16—H16	120.0
C15—C7—C8	119.5 (2)	C15—C16—H16	120.0
C9—C8—C7	104.95 (17)	C18—C17—C16	120.7 (3)
C9—C8—H8A	110.8	C18—C17—H17	119.6
C7—C8—H8A	110.8	C16—C17—H17	119.6
C9—C8—H8B	110.8	C19—C18—C17	119.8 (3)
C7—C8—H8B	110.8	C19—C18—H18	120.1
H8A—C8—H8B	108.8	C17—C18—H18	120.1
O1—C9—N1	123.2 (2)	C18—C19—C20	120.0 (3)
O1—C9—C8	122.3 (2)	C18—C19—H19	120.0
N1—C9—C8	114.4 (2)	C20—C19—H19	120.0
N1—C10—C11	112.4 (2)	C15—C20—C19	121.4 (3)
N1—C10—H10A	109.1	C15—C20—H20	119.3
C11—C10—H10A	109.1	C19—C20—H20	119.3
N1—C10—H10B	109.1		
			
C9—N1—C1—C2	−132.7 (2)	C9—N1—C10—C11	−108.0 (2)
C10—N1—C1—C2	52.3 (3)	C1—N1—C10—C11	67.3 (3)
C9—N1—C1—C6	49.8 (3)	C12—N3—C11—C10	−137.1 (2)
C10—N1—C1—C6	−125.2 (2)	C13—N3—C11—C10	66.2 (3)
C6—C1—C2—C3	0.7 (4)	N1—C10—C11—N3	57.5 (3)
N1—C1—C2—C3	−176.8 (3)	C14—O3—C12—O2	176.3 (3)
C1—C2—C3—C4	0.7 (5)	C14—O3—C12—N3	−4.3 (3)
C2—C3—C4—C5	−0.6 (4)	C11—N3—C12—O2	12.8 (4)
C3—C4—C5—C6	−1.0 (4)	C13—N3—C12—O2	172.0 (3)
C2—C1—C6—C5	−2.2 (3)	C11—N3—C12—O3	−166.5 (2)
N1—C1—C6—C5	175.2 (2)	C13—N3—C12—O3	−7.3 (3)
C2—C1—C6—N2	−179.1 (2)	C12—N3—C13—C14	14.8 (3)
N1—C1—C6—N2	−1.6 (3)	C11—N3—C13—C14	173.5 (2)
C4—C5—C6—C1	2.4 (4)	C12—O3—C14—C13	13.4 (3)
C4—C5—C6—N2	179.5 (2)	N3—C13—C14—O3	−16.4 (3)
C7—N2—C6—C1	−42.9 (3)	N2—C7—C15—C20	161.8 (2)
C7—N2—C6—C5	140.2 (2)	C8—C7—C15—C20	−22.5 (3)
C6—N2—C7—C15	173.44 (19)	N2—C7—C15—C16	−19.4 (3)
C6—N2—C7—C8	−2.2 (3)	C8—C7—C15—C16	156.3 (2)
N2—C7—C8—C9	76.5 (3)	C20—C15—C16—C17	0.4 (4)
C15—C7—C8—C9	−99.1 (2)	C7—C15—C16—C17	−178.4 (2)
C1—N1—C9—O1	178.2 (2)	C15—C16—C17—C18	0.2 (4)
C10—N1—C9—O1	−6.8 (3)	C16—C17—C18—C19	−1.3 (4)
C1—N1—C9—C8	−5.5 (3)	C17—C18—C19—C20	1.8 (5)
C10—N1—C9—C8	169.45 (19)	C16—C15—C20—C19	0.1 (4)
C7—C8—C9—O1	107.4 (3)	C7—C15—C20—C19	178.9 (3)
C7—C8—C9—N1	−68.8 (2)	C18—C19—C20—C15	−1.2 (5)
